# Transcriptome profiles of *Streptomyces clavuligerus* strains producing different titers of clavulanic acid

**DOI:** 10.1038/s41597-023-02727-6

**Published:** 2023-11-16

**Authors:** Junpyo Gong, Jeong Sang Yi, Hang Su Cho, Chang Hun Shin, Hyung-Jin Won, Byung-Kwan Cho, Minsoo Noh, Yeo Joon Yoon

**Affiliations:** 1 Natural Products Research Institute, College of Pharmacy, Seoul National University, Seoul, 08826 Republic of Korea; 2 Research Institute of CKD BiO, Ansan, 15604 Republic of Korea; 3 Department of Biological Sciences and KI for the BioCentury, Korea Advanced Institute of Science and Technology, Daejeon, 34141 Republic of Korea

**Keywords:** Gene expression, Data acquisition

## Abstract

*Streptomyces clavuligerus* NRRL 3585 is a native producer of clavulanic acid (CA), a clinically used β-lactamase inhibitor, and is widely used as an industrial strain for the production of antibiotics. Selective random mutagenesis has successfully generated the improved CA-producing *S. clavuligerus* mutant strains as well as the strain with the loss of CA biosynthesis. To understand the molecular mechanisms associated with the improved CA-production potential, genome-scale RNA-sequencing-based transcriptional data were obtained for the wild-type *S. clavuligerus* strain and its three mutant strains. Total RNA samples for each strain were collected across four different growth stages, and all 32 sequencing data points exhibited an average Phred score of 36. The high-quality genome-scale transcriptional profile of *S. clavuligerus* strains with varied CA biosynthetic potential provides valuable insights and new opportunities for discovering efficient metabolic engineering strategies for the development of improved industrial strains.

## Background & Summary

*Streptomyces* species are Gram-positive microorganisms that play a significant role in the production of valuable secondary metabolites such as antibiotics, anticancer drugs, and pesticides. *S. clavuligerus* is a native producer of clavulanic acid (CA) and cephamycin and is used as an industrial strain for the production of these compounds^1^. CA, a widely used β-lactamase inhibitor, is a major active component in the extensively prescribed antibiotic, amoxicillin-containing Augmentin^TM^. The β-lactam moiety of amoxicillin covalently binds to the bacterial proteins responsible for crosslinking peptidoglycan precursors that are essential for bacterial cell-wall formation^2^. However, many pathogenic bacteria can easily acquire resistance against β-lactam antibiotics such as amoxicillin by expressing β-lactamases^3^. Notably, CA has no direct antibiotic activity but can inhibit β-lactamases and thus restore β-lactam (i.e. amoxicillin) sensitivity in β-lactamase expressing strains.

**Fig. 1:**
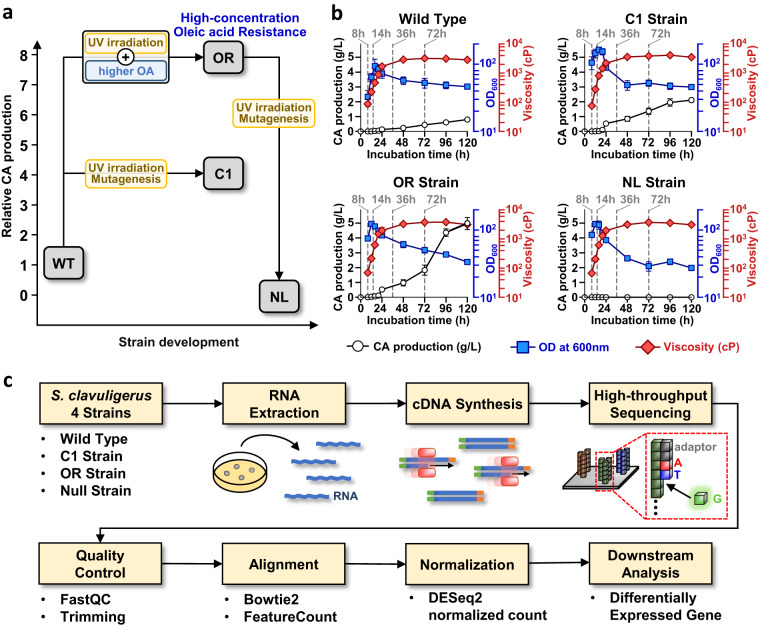
Experimental design and workflow. (**a**) Schematic diagram of the strain development process for the four *S. clavuligerus* strains, WT, C1, OR, and NL. (**b**) Biomass parameters measured as viscosity and OD, and CA production of the four *S. clavuligerus* strains. Sampling time points for RNA extraction is indicated by gray dashed lines. (**c**) Workflow of RNA-seq and data analysis.

Random mutagenesis using ultraviolet (UV) irradiation or chemical mutagens is an efficient strategy for improving the production yields of desired secondary metabolites, including CA, in diverse bacterial strains^4–6^. Recently, we generated improved CA-producing mutant strains from wild type (WT) *S. clavuligerus* NRRL 3585 through UV irradiation-induced random mutagenesis or metabolic engineering (Fig. 1a). For example, the *S. clavuligerus* C1 mutant strain (C1) generated through UV irradiation-induced mutagenesis exhibited an approximately two-to-three-fold increase in CA production compared to that of the WT strain. In addition, oleic acid (OA) can be an optimal carbon source for CA production; however, high OA concentrations in culture conditions inhibit the growth of *S. clavuligerus* strains. When bacteria were again challenged with UV irradiation in presence of high OA levels, the OA-resistant *S. clavuligerus* OR strain (OR) was selected^7^. The OR strain produces about eight-fold as much CA as that of the WT strain^8^. Notably, upon subjecting the OR strain to further UV irradiation, a null *S. clavuligerus* mutant strain (NL) with complete loss of CA biosynthetic activity was obtained. Although UV irradiation-induced random mutagenesis effectively generates diverse mutant strains with varied CA biosynthetic potentials, it is difficult to explain the genetic and molecular mechanisms simply by whole-genome sequencing of the mutated strains because genetic mutations occur at multiple loci in the genome. Transcriptional profile studies have therefore been suggested to compensate for the limitations of whole-genome sequencing^9,10^.

To understand the molecular mechanisms underlying the increased or decreased CA-producing capabilities, comparative transcriptional profiles of the four *S. clavuligerus* strains were analyzed. Genome-scale RNA-sequencing (RNA-seq) data for the four *S. clavuligerus* strains, WT, C1, OR, and NL strain were generated in this study (Fig. 1). RNA samples for each strain were extracted in biological duplicates at four different time points based on CA production: 8, 14, 36, and 72 h, where 8 and 14 h correspond to before and right after CA production begins, respectively, and 36 and 72 h to the time points where CA is actively produced. Consequently, we generated a RNA-seq dataset consisting of 32 samples, with sequence reads in the transcriptional samples ranging from 10.69 to 38.68 million. These data can provide valuable insights into novel methods for enhancing CA production ability of industrial strains through rational or targeted metabolic engineering strategies.

## Methods

### Strain, culture condition, and CA quantification

In this study, four strains of *S. clavuligerus*, namely WT, C1, OR, and NL, were used (Fig. 1a,b). *S. clavuligerus* NRRL 3585 was purchased from American Type Culture Collection. C1 and OR are UV irradiation-induced mutants of *S. clavuligerus* NRRL 3585 possessing a greater CA production ability than the WT. OR exhibits resistance to high OA concentrations, whereas C1 does not. The NL strain is a UV irradiation-induced mutant derived from the OR strain but lacks the ability to produce CA. Spore stocks of *S. clavuligerus* NRRL 3585 and its mutant strains, C1, OR, and NL, were maintained in 25% glycerol at −80 °C. The inoculation of *Streptomyces* spores and main incubation conditions followed the methodology outlined in a previous study^8^. In brief, spores of the *S. clavuligerus* strains were inoculated into 20 mL of seed medium in a 100 mL baffled flask containing 20 g/L of starch, 30 g/L of soy flour, 23 g/L of triolein, and 1.2 g/L of phosphate. Subsequently, 0.8 mL of the seed culture broth was transferred to a 50 mL production medium, which included 10 g/L of starch, 20 g/L of soy flour, 10.5 g/L of 4-morpholinepropanesulfonic acid, 23 g/L of triolein, and 1.2 g/L of phosphate, along with 1 mL of trace elements containing 3 g of iron (III) chloride hexahydrate, 0.5 g of copper (II) chloride dihydrate, 0.5 g of zinc chloride, and 0.5 g of manganese sulfate monohydrate in 1 L of distilled water, and 1 mL/L of antifoam in a 500 mL baffled Erlenmeyer flask. Cell growth was measured in terms of various biomass parameters due to complexities in the CA production medium^11^. Viscosity and optical density (OD) were used as biomass parameters to calculate specific cell growth as described in our previous studies (Fig. 1b)^8^. OD was measured at 600 nm. Viscosity was measured with viscometer equipped with a sample adaptor (Model DV-E, Ametek Brookfield, USA) as previously described^8^. Samples for RNA extraction were collected based on the CA production at four different time points (8, 14, 36, and 72 h). Two replicates were harvested at each time points to serve as biological replicates. For CA quantification, 0.1 mL of culture broth was mixed with 20 mM acetate buffer at pH 6.6. After removal of insoluble materials with 0.25 μm nylon syringe filter, it was analyzed with Waters 600 high performance liquid chromatography (HPLC) system equipped with a multisolvent delivery pump, a controller, and a photodiode array detector 2996 for CA detection (Waters 600 model, Waters, USA). The analysis was conducted with Hypersil^TM^ BDS C18 HPLC column, particle size of 5 μm with 4.6 × 250 mm dimensions (Thermo Fisher, USA), 14 min isocratic method using 16.6 mM NaH_2_PO_4_ in methanol (86:14, v/v), flow rate of 1 mL/min, and UV observance at 238 nm. CA standard was used for quantification of CA production. All reagents for which no specific vendor information has been provided were obtained from Sigma-Aldrich (USA).

### RNA extraction

The cells were harvested and washed with polysome buffer (140 mM NaCl, 20 mM Tris-HCl pH 7.5, and 5 mM MgCl_2_). Cell pellets were resuspended in lysis buffer (0.3 M sodium acetate pH 5.2, 10 mM EDTA, and 1% Triton X-100). The cell suspension was frozen using liquid nitrogen and then subjected to physical lysis by grinding with a mortar and pestle. The supernatant collected by centrifugation of the cell lysate at 4 °C for 10 min at 16000 × g was either used for RNA extraction, or stored at −80 °C. The supernatants were mixed with equal volumes of phenol, chloroform, and isoamyl alcohol (25:24:1) and subjected to centrifugation. After centrifugation, RNA was extracted from the aqueous layer and precipitated using ethanol.

### cDNA Library preparation and sequencing

The RNA samples were treated with DNase I (New England Biolabs, USA) to remove any DNA contamination. cDNA library preparation, quality assessment, and RNA sequencing were performed by Macrogen Inc. (Republic of Korea). A TruSeq stranded mRNA (Illumina, USA) preparation kit was used as per the manufacturer’s protocol, for cDNA library preparation. The cDNA library was sequenced using the Illumina NovaSeq6000 platform.

### Data processing of RNA-Seq reads

For data analysis, Trimmomatic v0.39 was employed to eliminate low-quality reads from the raw sequencing data in single-end mode using Phred + 33 quality score encoding^12^. The following steps were applied: removing leading and trailing bases with a quality score 3 or less, and sliding window trimming with a window size of four bases. If the average quality within the window dropped below 15, bases within the window were removed. Reads below the minimum length threshold of 36 bases were discarded. To ensure quality control of the RNA-seq reads before and after trimming, FastQC (https://www.bioinformatics.babraham.ac.uk/projects/fastqc/) v0.11.9 was used. Subsequently, the resulting trimmed single-end reads were aligned to the reference genome using Bowtie2 (v.2.3.4.3) with the parameters “–D 20 –R 3 –N 0 –L 20 –i S,1,0.50”^13^. The reference genome accession numbers for *S. clavuligerus* are NZ_CP027858.1, for the chromosome, and NZ_CP027859.1 for the plasmid, respectively, and both are available at NCBI Assembly GCA_005519465.1^14^. The mapped data were then processed to obtain expression count data using FeatureCounts v2.0.3^15^.

### Time-course gene expression changes and principal component analysis

**Fig. 2:**
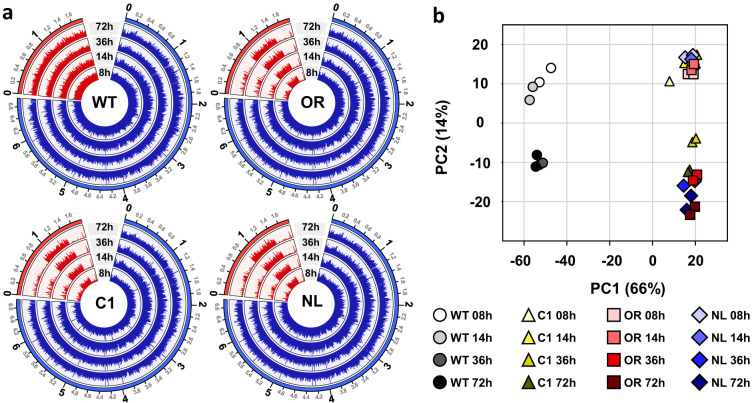
(**a**) Circos plots were used to visualize the RNA-seq data. The outermost circle represents the reference linear chromosome (blue) and the plasmid (red). Numbers on the outermost circle indicate chromosomal positions in Mb. The inner four tracks display bar plots based on the mean normalized gene expression counts for each biological duplicate sample at different growth phases. (**b**) The results of PCA on the gene expression profiles of each sample are visualized.

The expression count data for each gene were normalized using the DESeq2 package (version 1.40.1) in R (version 4.3.0)^16^. A circos plot was generated using the Circlize package (version 0.4.15) in R (Fig. 2a)^17^. Genes in the reference genome were sorted based on their positions on the chromosome. Each bar plot was generated based on the mean normalized gene expression counts from duplicate samples. Notably, the circos plot suggested that large deletions in the plasmid may have occurred in the mutant strains C1, OR, and NL. Principal component analysis (PCA) was performed on the samples, using the plotPCA function with the parameter “ntop” set to 300 in DESeq2 (Fig. 2b).

### Identification of differentially expressed genes

**Fig. 3:**
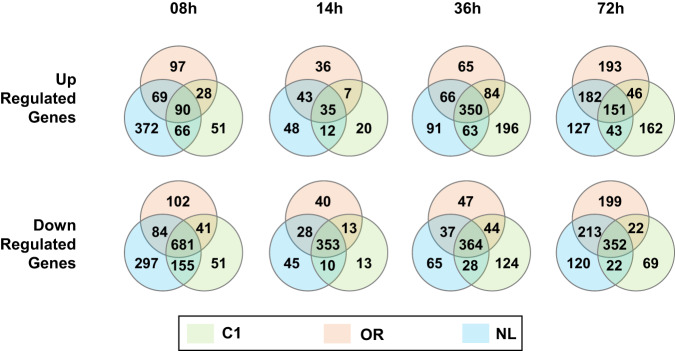
The Venn diagram analysis depicts the comparison of up-regulated and down-regulated DEGs in RNA-seq results of each *S. clavuligerus* mutant strain at the corresponding timepoint compared to the WT. The DEGs were identified using DESeq2 with a FDR cutoff of <0.001 and an absolute value of minimum fold change of >1^18^.

Differentially expressed genes (DEGs) in the three *S. clavuligerus* NRRL 3585 mutant strains were analyzed based on the WT transcriptome data (Fig. 3). The DEGs were identified using the DESeq2 library in R, with a false discovery rate (FDR) cutoff of <0.001 and a log fold expression change greater than 1 or less than −1. For each *S. clavuligerus* strain, the up-regulated and down-regulated DEGs compared to those of the wild-type, at corresponding time points were identified^18^.

## Data Records

All RNA-seq raw read FASTQ files were deposited in the Sequence Read Archive (SRA) of the National Center for Biotechnology Information (NCBI) under accession number SRP453950^19^. The DEGs for each comparison were deposited at the Figshare database^18^.

## Technical Validation

### Validation of RNA sequencing reads

**Fig. 4:**
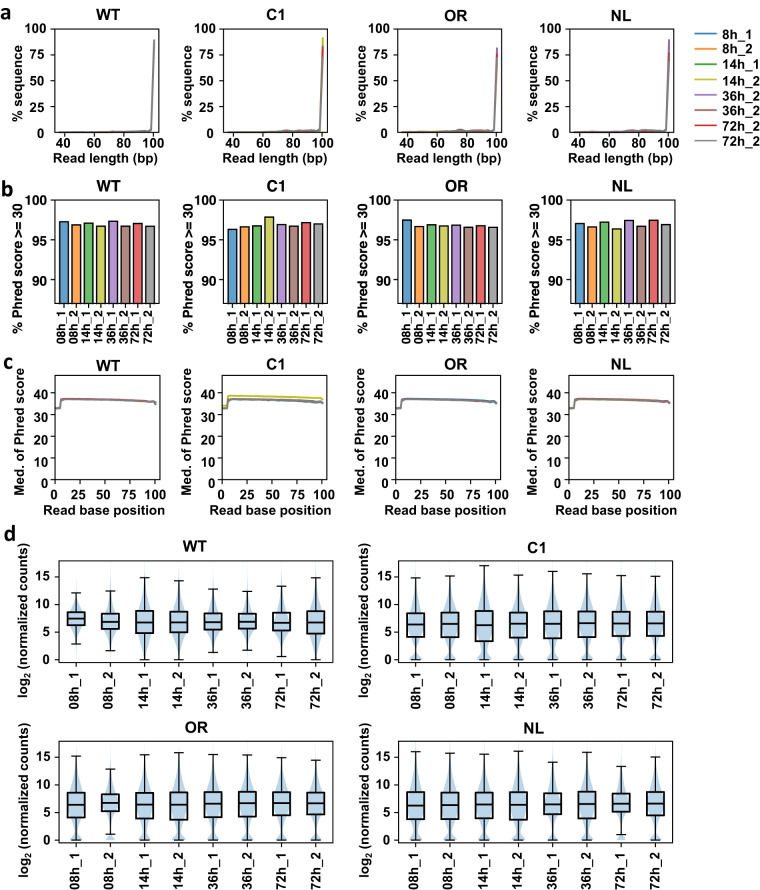
Technical Validations of RNA-seq samples from four different growth phases of four strains of *S. clavuligerus*. (**a**) The analysis of read length distribution was performed on trimmed reads obtained from RNA-seq samples of four different strains. For each growth phase, there were two replicates indicated by suffixes “_1” or “_2” after the time point. (**b**) The barplots depict the percentage of reads with a Phred score of 30 or higher for each RNA-seq sample. The average Phred scores of the reads after trimming were analyzed across the RNA-seq samples of the *S. clavuligerus* strains during various growth phases. (**c**) In the RNA-seq samples of four strains, the distribution of median Phred quality scores at each base position was examined. (**d**) The log_2_ normalized expression counts obtained from the DESeq2 package in the RNA-seq samples of the *S. clavuligerus* strains were visualized through violin and box plots.

A total of 32 RNA-seq libraries of *S. clavuligerus* NRRL 3585 and its mutant strains were generated. The data included NGS libraries generated at four different growth phases with biological duplicates (Fig. 1). FastQC was utilized for the quality assessment of Illumina RNA-seq reads, which indicated high sequencing quality, with an average of 99.16% of the trimmed reads remaining after quality scoring and nucleotide length trimming. After trimming, sequencing resulted in 10.60 to 38.43 million reads per library (Table 1). The RNA-seq samples had an average read length of approximately 100 bp (Fig. 4a). All RNA-seq data exhibited an average Phred score of 36 or higher, suggesting base-calling error probabilities lower than 10^−3^ at a minimum (Fig. 4b,c)^20,21^. Across all samples, an average of 96.9% of reads had a Phred score of 30 or higher. Based on these quality validation results, we confirmed the quality of all the obtained RNA sequencing reads prior to subsequent downstream analysis.

**Table 1:** Overall statistics of RNA-Seq data.

Species	Growth phase	Replicate	Raw reads	Total reads after trimming	Trimming survived (%)	Number of successfully assigned reads	Successfully assigned reads (%)
Wild Type	8 h	1	10,688,020	10,599,345	99.17	4,545,021	42.9
	8 h	2	21,382,700	21,255,774	99.41	14,280,348	67.2
	14 h	1	20,688,602	20,537,239	99.27	16,769,944	81.7
	14 h	2	20,485,371	20,347,368	99.33	15,752,626	77.4
	36 h	1	21,207,265	21,067,920	99.34	18,185,024	86.3
	36 h	2	16,055,799	15,918,218	99.14	12,365,589	77.7
	72 h	1	18,053,444	17,887,749	99.08	15,070,565	84.3
	72 h	2	18,849,869	18,714,674	99.28	11,838,693	63.3
C1	8 h	1	17,878,954	17,749,776	99.28	16,866,814	95.0
	8 h	2	38,681,553	38,429,975	99.26	27,332,772	71.1
	14 h	1	16,002,400	15,894,890	99.35	11,782,965	74.1
	14 h	2	19,131,312	19,090,159	99.14	14,431,006	75.6
	36 h	1	27,373,796	27,171,153	99.33	18,947,973	69.7
	36 h	2	26,262,952	26,038,184	99.39	17,223,746	66.1
	72 h	1	18,729,109	18,614,927	99.78	16,458,938	88.4
	72 h	2	17,607,196	17,479,014	99.27	16,381,852	93.7
OR	8 h	1	18,125,732	17,988,642	99.24	8,900,653	49.5
	8 h	2	18,642,529	18,305,390	98.19	9,032,882	49.3
	14 h	1	21,654,131	21,494,581	99.26	15,382,611	71.6
	14 h	2	18,416,942	18,210,767	98.88	10,827,689	59.5
	36 h	1	18,380,203	18,259,525	99.34	14,055,842	77.0
	36 h	2	18,618,929	18,387,729	98.76	14,543,760	79.1
	72 h	1	18,401,954	18,275,719	99.31	15,995,929	87.5
	72 h	2	19,001,097	18,823,218	99.06	16,471,929	87.5
NL	8 h	1	17,187,817	17,022,033	99.04	12,515,865	73.5
	8 h	2	17,534,934	17,399,566	99.23	11,941,694	68.6
	14 h	1	17,126,158	17,008,249	99.31	12,338,181	72.5
	14 h	2	18,075,332	17,690,452	97.87	11,323,309	64.0
	36 h	1	18,676,917	18,554,429	99.34	10,874,238	58.6
	36 h	2	15,683,592	15,545,230	99.12	13,122,474	84.4
	72 h	1	19,637,499	19,476,479	99.18	17,453,398	89.6
	72 h	2	11,755,510	11,645,576	99.06	10,373,505	89.1

### Evaluation of transcriptome data

The reads were aligned to the reference genome, resulting in an average mapping rate of 74.24% for RNA-seq, indicating a substantial proportion of mapped gene reads. The distribution of log_2_ (DESeq normalized count +1) exhibited a wide range, from 0 to 18 (Fig. 4d). Transcriptome profile visualization revealed a distinct pattern highlighting the differences between the WT and randomly mutated strains (Fig. 2a). PCA was performed to ensure the reproducibility of the biological duplicates. Overall, the plots demonstrated high reproducibility across all replicates (Fig. 2b). The DEGs of the three mutant strains were compared to those of the WT strain to evaluate the utility of transcriptome data in unraveling the molecular mechanism underlying the high CA production ability (Fig. 3). Each randomly mutated strain with different CA biosynthetic ability exhibited both, common and distinct DEGs at various time points. This suggests that the comparative analysis of transcriptome data can potentially uncover efficient strategies for enhancing CA productivity thereby producing improved industrial strains.

## Data Availability

All bioinformatic tools utilized in this study, along with their respective parameters, are clearly described in “Methods” section. In instances where specific parameters for the software were not specified, default parameters were employed as recommended by the developer.
